# Performance of Elite Women's Singles Badminton Players: The Influence of Left-Handed Players

**DOI:** 10.5114/jhk/172783

**Published:** 2023-11-28

**Authors:** Yibo Zhang, Bo Leng

**Affiliations:** 1 College of Physical Education, Taiyuan University of Technology, Taiyuan Shanxi, China.; 2 College of Competitive Sport, Beijing Sport University, Beijing, China.

**Keywords:** different game formats, notational analysis, women's singles match, technique and tactics

## Abstract

The purpose of the study was to analyze the influence of left-handed athletes on the opponent (right-handed players) of elite badminton women's singles. The study selected a total of 40 women's singles matches played by elite female players: twenty matches (42 games, n = 42) were played between left-handed and right-handed players and twenty matches (44 games, n = 44) were played between two right-handed players. There were significant (p < 0.05) differences in hitting positions, techniques, routes and landing points. No significant (p > 0.05) differences were found in scores per game and frequency distribution of rally outcomes. In conclusion, the details of technical and tactical application were different in two game forms, the main impact of the left-handed player on the opponent's (right-handed player) game was a decrease in the opponent's stroke in the overhead, an increase in the number of drives, predominance of small slashes and a decrease in big slashes.

## Introduction

According to sport data, left-handed athletes are more likely to be in the high echelons of interactive professional sports like badminton, tennis and fencing, but not non-interactive professional sports such as darts and snooker (Harris, 2016; Loffing, 2017; Witkowscki et al., 2019). However, factors that either increase or decrease the advantages of performing left-handed in interactive contests have not yet been definitively determined. The literature focuses primarily on possible explanations for this phenomenon (Grouios, 2000, 2004). The first is based on the innate superiority hypothesis, which states that left-handed people have a psychological advantage; second, that they possess a tactical or a strategic advantage (the hypothesis of the strategic advantage) (Grouios, 2000; Loffing et al., 2012).

Although the importance of left-handers has been recognized for a long time, there are times when research needs to avoid left-handers. In some studies, left-handers are left out of scientific research samples in order to reduce heterogeneity of participants (Nooijer et al., 2016). This is true for studies in neuroscience and neurogenetics (Willems et al., 2014), but also in psychology, linguistics, and human movement sciences such exclusions can occur. They are, unfortunately, still underrepresented (often absent altogether) in neuroimaging studies, which is understandable, given the high testing cost. Left-handers might be a minority, but the differences between left- and right-handers may prove valuable in our understanding of cognition.

Badminton is a competitive racket sport between two (single) or four (double) opponents. One of the particularities of badminton is that it is played with a shuttlecock, which gives it different characteristics within racket games. A match is played to the best of 3 sets, and a set is won when a player reaches 21 points, with a difference of, at least, 2 points. Every point won by a player gives the privilege to serve the next point. On even points, the serve is done from the right zone, and on odd points, it is performed from the left zone to the other side of the court (Galeano et al., 2022). Badminton is a dynamic, interactive racket sport that requires players to have excellent open skills. In general, sports may be categorized into two types: open skill and closed skill sports. Open skill sports are defined as those in which players are required to react in a dynamically changing, unpredictable and externally-paced environment. e.g., basketball, tennis, fencing etc. Badminton is a sport with a wide variety of movements and shuttlecock shots (Ghosh, 2008; Hong, 2000) and many repetitive game actions of short duration but high intensity, which clearly distinguishes between sequences of episodes of work and breaks (Cabello et al., 2004) that will determine its temporal structure.

Although strength, speed, and endurance are very important, the application and the outcome of technical-tactical actions are crucial to players' ability to outperform their opponents in dyadic interactions. Therefore, notational analysis of the key technical-tactical performance indicators that are linked to a positive result can provide practitioners with useful references on how to adjust training and match strategy accordingly and raise a player's competitive level (Hughes et al., 2002). Technical, physical, and temporal variables have been widely used in previous studies to interpret and quantify the match characteristics of badminton players, and the technical aspect has gained the most interest of the academic community, especially the singles modality (Gomez et al., 2020; Pablo et al., 2014; Sheng et al., 2022). New methods such as Markov chains, network science, classification tree analysis, dynamic analysis, etc., are being increasingly used to analyze performance of badminton players (Galeano et al., 2022; Gómez-Ruano, 2020; Gómez, 2021, 2020).

In studies about left-handed athletes, its chief benefit is to help understand why left-handers have the advantage. In the study of badminton techniques and tactics, sometimes researchers leave the left- and right-handed samples undifferentiated. However, putting the left- and right-handed athlete samples together affects the accuracy of the results of analysis. This may be related with the fact that the number of matches played by left-handed racket holders is smaller and more matches are played by the same left-handed player. The impact of the opponent’s handedness on performance, however, seems to be underrated in previous research (Yu et al., 2022). Studies of left-handed athletes in interactive sports provide clues for further investigation. The relationship between the handedness of rivals determines how players behave technically and tactically.

Badminton matches can be considered from the perspective of two formats, i.e., games between two right-handers (R1-R2) and between players of opposite handedness (R1-L). Therefore, in order to better understand the complexities of interacting performance factors in badminton match-play, the present study attempted to determine technical-tactical patterns that distinguished between the performance of elite women's badminton players in two different singles game formats. This was performed by observing multiple performance indicators of every stroke to make comparisons between the same groups (right-handed players) competing in different game formats. Thus, differences among particular contexts of play for the same right-handed player against different opponents were analyzed.

## Methods

### Participants and General Procedure

Adopting an observational study design, the study selected a total of 40 women's singles matches (86 games) played by elite female players (world rankings in the top 30) and collected the detailed data of each stroke (n = 28703). Two kinds of games were considered. Twenty matches (R1-L: 42 games, n = 42) were played between left-handed and right-handed players and 20 matches (R1-R2: 44 games, n = 44) were played between two right-handed players. R1 was the same group of right-handed players and it included two athletes: Chen Yu Fei and Tai Tzu Ying. Approximately 20 matches were selected for each person. L was the same group of four left-handed players, namely Carolina Marin, He Bing Jiao, Aya Ohori, and Sayaka Takahashi. Approximately five matches were selected for each person. R2 was the same group of right-handed players, chosen to be ranked similarly to left-handed players. It totaled 20 matches.

Due to the timing and the design of the competition, matches were played with the current rally point scoring system, where the one who won the best of three games of 21 points was the winner. Given the category of the tournament, all participants were the best players in the world at that time. The women's singles matches were chosen for the study because the match samples were more readily available and the number of elite left-handed women singles players was relatively larger at that time. We believe that the top 30 athletes represented the highest performance level and there were four left-handed players in the women's singles.

Since there were no differences in notational structures between games in the same matches in previous studies, the game was chosen as the measurement unit (Abian-Vicen et al., 2013) in which we calculated the value of the variable. We believe that the game is the best structure for standardizing measurements and comparing them to other performance studies that have been performed in the past without limiting the number of games in the match (Pablo Abián et al., 2014).

### Materials

Official videos recorded by the Badminton World Federation (BWF) from 2018 to 2021 BWF World Tour (above the super 500 level) were used to carry out the analysis of the matches. It is worth mentioning that all measurements and observations were completed by the same investigator who had extensive training in the methods and procedure of this study. The analyst was instructed to watch the videos of the badminton matches and record his observations directly using the badminton games information recording system (Zhang et al., 2022). In the distinction of dominant hands, we classified players as right- or left-handed purely according to the hand holding the racquet.

The study focused on the same right-handed players playing against right- and left-handed athletes (two possible scenarios of play, R1-L: a right-handed vs. a left-handed player and R1-R2: a right-handed vs. a right-handed player). Therefore, differences among particular contexts of play for the same right-handed player against different opponents were evaluated. In two game formats, R1 was the same group of right-handed players. Moreover, L and R2 groups included women's singles players of a similar competition level, yet with different racket holders.

### Data Collection and Reliability

Independent variables were the game formats (the same right-handed player against different opponents), while dependent variables included the results of game scores and shots (total scores per game, shots per game, Table 1). The percentages obtained for each type of game variables were also analyzed: rally outcome, positions, techniques, routes, landing points (see definitions in Tables 2–6).

**Table 1:** Comparison of game scores and shots.

Scores and shots	R1-L *M ± SD (%)*	R1-R2 *M ± SD (%)*	*t*	*p*	Cohen's *d*
Total scores per game	35.14 ± 3.57	36.55 ± 3.97	−1.719	0.089	0.373
Shots per game	314.17 ± 68.07	352.30 ± 86.03	−2.272	0.025	0.490

**Table 2:** Frequency distribution of the rally outcome.

Rally Outcome	R1 (R1-L) *M ± SD (%)*	R1 (R1-R2) *M ± SD (%)*	*t*	*p*	Cohen's *d*
Winner	36.42 ± 15.78	35.27 ± 10.88	0.391	0.694	0.084
Forced error	25.29 ± 12.15	24.33 ± 11.77	0.369	0.713	0.080
Unforced error	38.30 ± 11.65	40.40 ± 11.51	−0.841	0.403	0.181

Two professional badminton analysts, both with more than ten years of experience in badminton match analysis, participated in data collection. Prior to the formal procedure, a training session was held to master the computer program. Afterwards, a preliminary study was conducted on two randomly chosen matches (five games) to test intra-observer and inter-observer reliability. Matches were randomly selected from the same overall sample (two R1-L games and three R1-R2 games).

The inter-observer reliability was evaluated by comparing the results of the second analyst to the initial observation of the leading analyst. The inter-observer reliability was measured by the calculation of the Cohen’s Kappa value (Cohen, 1960). The inter-observer reliability for match analysis was assessed as good to very good (all Kappa ≥ 0.81) from a selection (n = 5) of randomly selected matches (Altman, 1991). Kappa values of inter-observer reliability were 1.000, 0.841, 0.839,0.830, 0.835 and 0.833 for the number of shots, rally outcomes, positions, techniques, routes, and landing points variables, respectively.

### Statistical Analysis

The following software programs were used: Microsoft Excel spreadsheet (Microsoft, Spain) to store the results and IBM SPSS 25.0 (IBM. Corp. Armonk, NY) to perform statistical calculations using descriptive and inferential statistical tests and to calculate means, standard deviations and ranges. Initially, normality was tested in all variables with the Shapiro-Wilk test. After that, the Student’s *t* test for independent samples was used to establish the differences in normally distributed variables between the two game formats. The criterion for statistical significance was set at *p* < 0.05. All the data are presented as mean ± standard deviation. In addition, Cohen's *d* was used to calculate ES with interpretations based on the following values: 0.20 = small effect, 0.50 = medium effect, and 0.80 = large effect (Lenhard et al., 2016).

## Results

### Scores and Shots per Game

Table 1 shows the descriptive statistics, results of the *t* test for total scores per game and shots per game of two game formats. No significant (*p* > 0.05) relationships were identified for total scores per game for both R1-L and R1-R2 women´s singles games. However, shots per game of the R1-R2 format were significantly higher than those of the R1-L format with a small effect size (*p* < 0.05, ES = 0.490).

### Rally Outcome Distribution

The frequency distribution of the rally outcome for each cluster is shown in Table 2. Regardless of the format, the unforced error was the most frequent last shot of the rally. No significant (all *p* > 0.05) differences were found in any of the rally outcome between R1-L and R1-R2 women´s singles games.

### Hitting Position Distribution

Table 3 displays the specific frequency distribution in relation to the hitting positions. No significant differences were found in forehand or backhand strokes between the game formats, yet a significant difference was observed in the frequency distribution of the overhead shot. Overhead shots of R1-R2 games were significantly higher than those of R1-L games with a small effect size (*p* < 0.05, ES = 0.492).

**Table 3:** Comparison of hitting positions.

Positions	R1 (R1-L) *M ± SD (%)*	R1 (R1-R2) *M ± SD (%)*	*t*	*p*	Cohen's *d*
Forehand	55.83 ± 8.72	55.92 ± 6.02	−0.056	0.956	0.012
Backhand	33.19 ± 8.64	31.07 ± 7.60	1.210	0.230	0.261
Overhead	10.98 ± 3.48	13.01 ± 6.66	−2.281	0.025	0.492

### Technique Distribution

Table 4 shows the frequency distribution of techniques of two game formats. There was no significant difference in the frequency distribution between most techniques (net drop, lift, cross-court net shot, push, kill and brush, block, clear, drop, smash, others: all *p* > 0.05). However, the number of drives of left-handed players was significantly higher than that of right-handed players with a small effect size (*p* < 0.05, ES = 0.444).

**Table 4:** Comparison of particular techniques.

Techniques	R1 (R1-L) *M ± SD (%)*	R1 (R1-R2) *M ± SD (%)*	*t*	*p*	Cohen's *d*
Spinning Shuttle and net drop	12.13 ± 4.39	12.31 ± 3.60	−0.203	0.839	0.044
Lift	24.78 ± 5.03	24.00 ± 4.66	0.743	0.460	0.160
Cross-Court Net Shot	2.86 ± 2.24	2.11 ± 1.53	1.802	0.076	0.389
Push	1.70 ± 1.17	1.55 ± 1.24	0.603	0.548	0.130
kill and brush	0.87 ± 0.76	0.90 ± 0.86	−0.195	0.846	0.042
Drive	3.56 ± 2.17	2.53 ± 2.46	2.059	0.043	0.444
Block	13.27 ± 4.37	12.60 ± 4.68	0.680	0.499	0.147
Clear	14.30 ± 5.20	16.32 ± 6.30	−1.615	0.110	0.348
Drop	10.75 ± 4.22	12.14 ± 4.49	−1.477	0.143	0.319
Smash	13.16 ± 4.89	12.30 ± 3.62	0.924	0.359	0.199
Others	2.62 ± 1.83	3.24 ± 1.86	1.568	0.121	0.338

### Hitting Route Distribution

The hitting route is an index with the most difference between the two game formats (Table 5). During hitting routes of women’s singles games, routes 2, 4, 5, and 6 of the R1-L game format were significantly (all *p* < 0.05) greater compared to the R1-R2 cluster. However, route 7 of R1-L was significantly (all *p* < 0.05) lower compared to the R1-R2 cluster. Medium effect sizes were noted for hitting routes 2 (ES = 0.508), 4 (ES = 0.561), 6 (ES = 0.563), and small effect sizes were noted for hitting route 5 (ES = 0.487).

**Table 5:** Comparison of hitting routes.

Routes		R1 (R1-L) *M ± SD (%)*	R1 (R1-R2) *M ± SD (%)*	*t*	*p*	Cohen's *d*
s+Left Court	1 (Straight)	25.78 ± 6.39	27.45 ± 5.39	−1.312	0.193	0.283
	2 (Small slash)	5.38 ± 2.86	4.08 ± 2.22	2.353	0.021	0.508
	3 (Big slash)	12.55 ± 4.70	11.48 ± 3.39	1.215	0.228	0.262
Centre Court	4 (Small slash)	6.08 ± 3.15	4.57 ± 2.12	2.599	0.011	0.561
	5 (Straight)	5c.69 ± 3.96	3.89 ± 3.42	2.256	0.027	0.487
	6 (Small slash)	6.77 ± 3.03	5.09 ± 2.93	2.609	0.011	0.563
						
Right Court	7 (Big slash)	9.31 ± 3.41	11.5 ± 4.17	−2.652	0.010	0.572
	8 (Small slash)	5.77 ± 2.61	6.67 ± 2.52	−1.625	0.108	0.351
	9 (Straight)	22.68 ± 5.76	25.28 ± 6.62	−1.941	0.056	0.419

### Landing Point Distribution

Table 6 shows the frequency distribution of landing points in the games. No significant differences (all *p* > 0.05) were found in forecourt landing points (1, 2, 3) between the game formats. The main differences were found in the frequency distribution of the midcourt and the backcourt. There were significantly more landing points 5 and 6 of left-handed compared to right-handed players with small or large effect sizes (*p* < 0.05, ES = 0.439; ES = 0.844). On the contrary, landing points 7 and 9 of left-handed players were significantly lower than those of right-handed players with small or moderate effect sizes (*p* < 0.05, ES = 0.458; ES = 0.550).

**Table 6:** Comparison of landing points.

Landing Points		R1 (R1-L) *M ± SD (%)*	R1 (R1-R2) *M ± SD (%)*	*t*	*p*	Cohen's *d*
Forecourt	1 (Left)	15.58 ± 4.00	16.42 ± 3.87	−0.990	0.325	0.214
	2 (Centre)	5.85 ± 3.02	5.04 ± 3.23	1.201	0.233	0.259
	3 (Right)	13.87 ± 3.64	14.11 ± 3.98	−0.297	0.767	0.064
Midcourt	4 (Left)	11.50 ± 4.16	11.14 ± 3.89	0.413	0.680	0.089
	5 (Centre)	5.76 ± 3.61	4.42 ± 2.41	2.035	0.045	0.439
	6 (Right)	12.44 ± 4.57	9.12 ± 3.22	3.914	0.000	0.844
Backcourt	7 (Left)	15.14 ± 5.12	17.48 ± 5.14	−2.121	0.037	0.458
	8 (Centre)	4.85 ± 2.50	4.23 ± 2.46	1.158	0.250	0.250
	9 (Right)	15.01 ± 5.09	18.03 ± 5.86	−2.550	0.013	0.550

## Discussion

The purpose of this study was to compare performance of right-handed elite women’s singles badminton players in different game formats. There were differences regarding technical and tactical aspects between athletes with different dominant hands, which directly affected the overall performance of the opponent's game. The present study focused on the same right-handed athletes playing against right- and left-handed players. Therefore, we could evaluate differences among particular contexts of play for the same right-handed players against different opponents.

The number of strokes by right-handers decreased significantly in games versus left-handers compared to games versus right-handers. With a little change in scores per game, there were about 40 fewer strokes per game when playing with a left-handed player. It confirmed that right-handed strokes are simpler to distinguish in interactive sports, which also means that it is easier to predict the stroke's direction. Additionally, the extremely large effect size highlights this effect's high significance (Hagemann, 2009). The average number of strokes per game was significantly lower due to the reduced predictability of right-handed players for left-handed players.

The main findings of this study showed no significant difference in frequency distribution of the rally outcome between the different games played. In elite badminton women's singles games, the difference in the opponent's racket holding hand does not affect the frequency distribution of the right-handed player's game score. The influence of the left-handed player on the opponent of the game does not lie in the final result of the stroke, but mainly in the opponent's technique and tactics used (definitions in Tables 3–6).

Difference in the hitting position is directly related to the opponent's hitting position and route. A game between players with different racket hands is very different from a game between two right-handed players in terms of space to hit the ball. In singles game, pressing the opponent's overhead is an important tactic, and right-handed players are able to return the ball more often to the opponent's (right-handed) overhead because a straight ball can be returned faster.

The position of the ball is to a certain extent determined by the use of hitting techniques. Since all aspects of the hitting elements are closely linked, changes in one aspect will cause changes in other aspects, thus affecting the technical and tactical performance of the whole game. When playing against left-handed players, there are significantly more drives, which is a reflection of the faster pace of play on both sides.

Florian Loffing's research concluded that a left-hander’s advantage is linked to the sports’ underlying time pressure in elite interactive ball games (Loffing, 2017). If the same point applies to badminton, the increase in drives may be more favorable for left-handed players to win, thus left-handed players performed more drives with their opponents during the game. The additional cognitive task (reacting to stimuli) could affect the time required for orienting and positioning the limbs, which in turn could result in lower velocities for right-handed players (Fasold et al., 2022). The more drives and the faster the tempo, the easier it is for the left-handed player to gain a time pressure advantage. Badminton is a unique sport that differs from other racket sports (i.e., tennis or paddle) due to the rapid responses required during high-intensity actions involving a shuttlecock flying at high speeds that does not make contact with the ground (i.e., similarly to volleyball) (Gómez, 2020).

Unlike the game between two right-handed players, the presence of a left-handed player directly changes the relative spatial position of the court to which the right-handed player is accustomed. This results in different hitting routes and landing points, and has an impact on the technique used. A straight line shot becomes a forehand-to-forehand and a backhand-to-backhand situation when a left-handed athlete plays against a right-handed one. The customary line of a right-handed player's forehand that presses and attacks the opponent's (right-handed) backhand area corresponds to his/her forehand when he/she plays badminton with a left-handed player. Attacking the left-handed backhand area needs to be achieved by hitting the ball diagonally, which imposes a longer flight distance and relatively more reaction time for the opponent. In the Chinese badminton team's summary, Cheng (2019) states that speed is the core of winning badminton games. The trajectory of the ball becomes longer, and the speed of the stroke is relatively lower, which is not favorable for right-handed players to win. Furthermore, changes of direction can be considered one of the most important athletic skills needed for a badminton player, which is favorable for left-handed players to win (Fernandez-Fernandez et al., 2022). Left-handed players have relatively more time to change direction when the ball travels along a longer trajectory.

The combination and variation of the hitting route and the landing point are core tactical aspects of badminton, tennis, table tennis and other (racket-holding) sports, and the use of technique is very closely related to the arc, direction and the landing point of the ball (Zhang et al., 2007). Depending on the position of the opponent's forehand and backhand, the women's singles player (right-handed) must adjust the hitting route and the landing point in order to achieve the corresponding tactical purpose.

### What Accounts for the Left-Hander’s Advantage?

Since the overall number of left-handed players is relatively small, left-handed players have more opportunities to play against right-handed players in training and competition, and are more adapted to the striking habits of right-handed players, while right-handed players are relatively less adapted to the way of play of left-handed athletes. The right-handed player's discomfort with the left-handed player and the active adjustment to the left-handed player lead to a change in the applied techniques and tactics. At the same time, it is worthy to note that Harris (2010) claims that at least some left-handers may have certain natural advantages. According to his overview, these advantages could be attributed to differences in motor control, attention, and the speed of transfer of primary-level sensory and motor information across the cerebral hemispheres.

In relation to the incidence in the normal population, left-handed athletes have been found more frequently at the elite level of duel-like interactive individual sports (e.g., fencing, table tennis, boxing) and team sports where one-on-one interactions between opponents are essential components of a game (e.g., baseball, cricket). Such overrepresentation is interpreted as indirect evidence for an advantage, which seems to occur only in interactive, but not in noninteractive sports such as darts, snooker or bowling (Loffing et al., 2016).

In interactive sports, athletes need to respond quickly and accurately to changing movement scenarios, and the response inhibition plays a critical role in this process. Research shows that left-handed athletes outperform right-handed ones in conflict perception and monitoring, but process slower than right-handed athletes (Gu et al., 2014). Hence, the left-handedness advantage may be based on their central processing ability advantage or it may be a “frequency-dependent” effect.

For example, both in their offensive and defensive actions, fencers produced more fixations to the armed hand and spent more time observing the armed hand in duels with a left-handed (vs. right-handed) opponent (Mateusz et al., 2020). Badminton is a process of control and counter-control between two sides, which is highly interactive and fast. Physiological and psychological differences between left- and right-handed players are important reasons for the different use of techniques and tactics. Badminton is a sport of control and counter-control between the two sides, which is highly interactive and fast, and differences in physiology and psychology between left- and right-handed players are important reasons for the different use of techniques and tactics. Therefore, when playing with left-handed athletes, techniques and tactics used by right-handed athletes will be different.

Experimental data showed that athletes had more difficulty predicting the movement intentions of left-handed than right-handed players in tennis and volleyball (Hagemann, 2009; Loffing et al., 2015), and that this perceptual cognitive difference provided left-handed players with an advantage, while video intervention training increased expectations for left-handedness and reduced the perceived advantage of left-handedness in interactive sports (Schorer et al., 2012). Ayala and Campbell (1974) suggest that frequency-dependent selection is a mechanism for the evolution of dominance. Increased training with left-handed players and real-life simulations of important left-handed opponents are definitely the most optimal ways to prepare for a match. Using a left-handed player as a training opponent will improve the adaptation to the left-handed player's stroke (Sampras, 1998).

## Conclusions

From these results, we can conclude that total scores per game and frequency distribution of the rally outcome are basically the same for two game formats, while the main difference is in the application of techniques and tactics. The presence of a left-handed player changes the familiar spatial position of the right-handed player, and different techniques and tactics used by the left-handed player change techniques and tactics used by their opponent (right-handed player). The main impact of the left-handed player on the opponent's (right-handed player) game includes decreases in the overhead opponent's strokes, increases in the number of drives, predominance of small slashes (Routes: 2, 4, 6) and decreases in big slashes (Route: 7). a decrease in the opponent's stroke in the overhead, an increase in the number of drives, predominance of small slashes and a decrease in big slashes.

Differences in the technical and tactical use of left-handed and right-handed players during a game can also change performance of the opponent, thus right-handed players should increase simulation or video training with left-handed players. At the same time, different racket hand players should be treated differently in badminton game performance, training and competition plan development to improve the accuracy of analysis and decision making.
